# A qualitative study on pharmacy policies toward over-the-counter syringe sales in a rural epicenter of US drug-related epidemics

**DOI:** 10.1186/s12954-021-00569-2

**Published:** 2022-01-08

**Authors:** Monica Fadanelli, Hannah L. F. Cooper, Patricia R. Freeman, April M. Ballard, Umed Ibragimov, April M. Young

**Affiliations:** 1 Emory University Rollins School of Public Health, 1518 Clifton Rd, Atlanta, GA 30322 USA; 2 University of Kentucky College of Public Health, Lexington, KY USA; 3 University of Kentucky College of Pharmacy, Lexington, KY USA

**Keywords:** People who inject drugs, Rural, Syringe sale, Pharmacy, Attitudes, HIV, HCV, Risk environment framework, Qualitative

## Abstract

**Background:**

Expanding access to sterile syringes in rural areas is vital, as injection-related epidemics expand beyond metropolitan areas globally. While pharmacies have potential to be an easily accessible source of sterile syringes, research in cities has identified moral, legal and ethical barriers that preclude over-the-counter (OTC) sales to people who inject drugs (PWID). The current study builds on prior urban-based research by elucidating (1) pharmacy OTC policies and (2) pharmacists’ rationale for, and barriers and facilitators to, OTC syringe sales in a US rural area hard hit by drug-related epidemics.

**Methods:**

We conducted 14 semi-structured interviews with pharmacists recruited from two Eastern Kentucky health districts. Interview domains included experiences with, and attitudes toward, selling OTC syringes to PWID. Constructivist grounded theory methods were used to analyze verbatim transcripts.

**Results:**

Most pharmacists operated “restrictive OTC” pharmacies (*n* = 8), where patients were required to have a prescription or proof of medical need to purchase a syringe. The remainder (*n* = 6) operated “open OTC” pharmacies, which allowed OTC syringe sales to most patients. Both groups believed their pharmacy policies protected their community and pharmacy from further drug-related harm, but diverging policies emerged because of stigma toward PWID, perceptions of Kentucky law, and belief OTC syringe sales were harmful rather than protective to the community.

**Conclusion:**

Our results suggest that restrictive OTC pharmacy policies are rooted in stigmatizing views of PWID. Anti-stigma education about substance use disorder (SUD), human immunodeficiency virus (HIV), and Hepatitis C (HCV) is likely needed to truly shift restrictive pharmacy policy.

## Introduction

Expanding access to sterile syringes in rural areas is vital as epidemics of injection-related human immunodeficiency virus (HIV) and hepatitis C (HCV) escalate outside metropolitan areas in the USA (US), Canada, China, and elsewhere [34, 45, 49]. Testifying to this expansion in the USA, the fastest-moving HIV outbreak ever identified in the USA was detected among people who inject drugs (PWID) in rural Indiana in 2015 [10], and a 2016 assessment conducted in response to this outbreak concluded that the counties at greatest risk for injection-related HIV and HCV outbreaks were “overwhelmingly” rural [45]. Subsequent epidemiologic data support this assessment. In the USA, two primarily rural states—Kentucky and West Virginia—were among the highest rates of reported acute HCV cases in 2018, about triple the national average rate of 1.2, at 3.7 and 3.8 per 100,000 persons, respectively [5].

Pharmacies may be valuable sources of sterile syringes in rural areas hard hit by injection-related epidemics. In many rural areas globally, pharmacies are plentiful, and pharmacists are one of the most accessible frontline health care providers; they are well positioned to provide care and advice, especially for vulnerable populations in limited resource settings [14, 21, 43]. In the USA, data indicate that 89% of Americans live within five miles of a pharmacy [2, 37]. In the USA, nationally, 20% of SSPs reported their primary location as rural, compared to 69% reported their primary location as urban and 9% reported their primary location as suburban [17, 18]. In rural areas that already have syringe service programs (SSPs), pharmacies may be vital complements to these existing programs: while SSPs may have limited operating hours, rural pharmacies may be open for upward of eight hours a day, five days a week [4]. These hours may enhance access to sterile syringes among rural PWID who work or have other time constraints that impact attendance, fear attending an SSP [22, 44], or due to local SSP closures [28, 29].

Research conducted in cities, however, has identified barriers to over-the-counter (OTC, i.e., without a prescription) pharmacy sales of syringes to PWID. In many countries, criminalization of possession and use of drugs and paraphernalia [16] or ambiguous or incongruent laws regulating syringe sales create apprehension among some pharmacists, curtailing OTC syringe sales [8, 9, 21]. While access to, and support for, OTC syringes has increased the USA in response to the HIV epidemic and opioid epidemic [24, 47], the USA still lags behind countries like the United Kingdom, France and Canada regarding decriminalizing syringe possession and OTC syringe sales [41]. Additionally, commercial and moral concerns among pharmacists can help fuel prohibitive or restrictive pharmacy-level OTC syringe policies; some pharmacists operating in urban areas express concern that their business may be disrupted by PWID, and/or worry about possible moral consequences of selling OTC syringes to people who will use them for illegal and possibly life-threatening behaviors [8, 9, 48]. As a result, research conducted in cities indicates that pharmacists act as gatekeepers [8, 9] and often establish their own prohibitive or restrictive policies, even when practicing in a jurisdiction that permits OTC syringe sales.

Few studies have explored this topic in rural areas. Most qualitative research on OTC syringe sales in rural areas has focused on PWID experiences with purchasing OTC syringes in pharmacies [22, 33] and hints at considerable pharmacy-based barriers to OTC sales. A landmark 2021 quantitative study conducted in three predominately rural US Appalachian states revealed significant differences in pharmacists’ behaviors and perceptions across state lines, suggesting that the political environment fosters some but not all pharmacist engagement in OTC syringe sales [20].

Here, we extend this past research to include community pharmacists practicing in two health districts in Appalachian Kentucky, a region at the heart of the US’ expanding rural opioid epidemic [45]. Interviewed pharmacists practiced in an ambiguous legal environment for OTC syringe sales at the time of data collection: state law permitted adult residents to purchase syringes OTC, but also required pharmacists to maintain a detailed OTC syringe sale log (Box 1). Pharmacists who did not follow these procedures could be charged, jailed or fined [25–27]; dispensing syringes outside an SSP (e.g., through a pharmacy) was not clearly legal (Box 1) [27]. Within this ambiguous legal rural context, the present study is designed to: (1) elucidate OTC syringe sale policies among pharmacists practicing in two rural health districts; (2) explore pharmacists’ rationales for their OTC syringe sale policies; and 3) understand pharmacists’ attitudes toward, and other barriers and facilitators, to OTC syringe sales.

**Box 1:** Excerpts of Kentucky revised statutes (KRS) from Kentucky law. The state of Kentucky has policies in place governing the sale and distribution of hypodermic syringes and definitions of what constitutes paraphernalia. Included are the current versions of each statue as well as the consequences for being charged and found guilty of violating these laws

KRS 217.177 Sale and disposal of hypodermic syringes or needles	KRS 218A.500 Definitions for KRS 218A.500 and 218A.510
(1) No person engaged in sales at retail shall display hypodermic syringes...in any portion of the place of business which is open or accessible to the public. (2) Every person engaged in sales of hypodermic syringes or needles at retail shall maintain a bound record in which shall be kept: (a) The name of the purchaser; and (b) The address of the purchaser; and (c) The quantity of syringes or needles purchased; and (d) The date of the sale; and (e) Planned use of such syringes or needles (3) Said record shall be maintained for a period of two years from the date of the sale and shall be available for inspection during business hours by any law enforcement officer, agent or employee of the Cabinet for Health and Family Services or Board of Pharmacy engaged in the enforcement of KRS Chapter 218A (4) No person shall present false identification… in obtaining or attempting to obtain any hypodermic syringe or needle (5) No person engaged in the retail sale of hypodermic syringes or needles shall: (a) Fail to keep the records required by this section…	(1) “Drug paraphernalia” means all equipment… which are used, intended for use, or designed for use in …injecting…or otherwise introducing into the human body a controlled substance in violation of this chapter. It includes but is not limited to: …(k) Hypodermic syringes, needles… (3) It is unlawful for any person to deliver, possess with intent to deliver… drug paraphernalia, knowing, or under circumstances where one reasonably should know, that it will be used to… inject…or otherwise introduce into the human body a controlled substance in violation of this chapter… (5) (a) This section shall not prohibit a local health department from operating a substance abuse treatment outreach program which allows participants to exchange hypodermic needles and syringes… (c) Items exchanged at the program shall not be deemed drug paraphernalia under this section while located at the program …(7) Any person who violates any provision of this section shall be guilty of a Class A misdemeanor

KRS 534.040 Fines for misdemeanors and violations	KRS 532.090 Sentence of imprisonment for misdemeanor
(1)…(a) For a Class A misdemeanor, five hundred dollars ($500) …	(1) For a Class A misdemeanor, the term shall not exceed twelve months…(2)…

## Methods

### Sample

The study area captured pharmacists practicing in two Eastern Kentucky health districts, which collectively span 12 counties. The current study is embedded within the broader CARE2HOPE project, a study of the risk environment for opioid use disorder [12], HIV, HCV, and overdose in two Eastern Kentucky health districts. We applied purposive sampling methods to recruit local pharmacists, seeking to recruit at least one community-based (i.e., non-hospital based) pharmacy in each county, and we sought representation from both independent and chain pharmacies. Research staff contact the pharmacists at these pharmacies via phone or in person and invited them to learn more about the study and consider taking part in the consent process and interviews.

### Data collection

After pharmacists consented, trained interviewers conducted one-on-one, semi-structured interviews in a private location inside each pharmacy. Each interview lasted one hour on average, and data were collected between February 2018–January 2019. The interview guide covered multiple domains, including experiences with, and attitudes toward, selling OTC syringes to PWID; insights into the local opioid epidemic and its drivers; and receptivity to pharmacy-based harm reduction initiatives. Pharmacists received a modest incentive ($10). Interviews were audiotaped and transcribed verbatim.

### Analysis

Constructivist grounded theory methods were used to analyze the transcripts [6, 7]. These methods recognize that individuals enter the field and analyze data with pre-existing assumptions and theories, which can act as “sensitizing constructs.” Charmaz ([6], p. 515) defined sensitizing constructs as ways of, “…seeing, organizing, and understanding experience.” The analysis was informed by two sensitizing constructs: (1) past literature on barriers and facilitators of OTC syringe sales like pharmacist stigma toward PWID, education, and the political environment and (2) environment types and levels of influence as defined by the Risk Environment Framework (REF). REF conceptualizes the risk environment as the “the space where a variety of factors interact to increase or decrease the chance of harm occurring” ([39], p. 193). REF envisions four environment types, the social, political, economic, and physical environments, each of which operate across micro- (immediate), meso- (institutional) and macro- (societal) levels [38, 40]. These different environment types and levels of influence interact with one another in dynamic ways to produce or reduce drug-related risks and outcomes [40]. Subsequent iterations of REF have added two additional environment types: the health care and law enforcement intervention environment and the epidemiologic environment [11, 13]. We situated our grounded theory categories within the context of the Risk Environment Framework (REF). Therefore, we classified our outcome, pharmacy OTC syringe sales, as a meso-level characteristic of the local health care/criminal justice intervention environment.

**Fig. 1:**
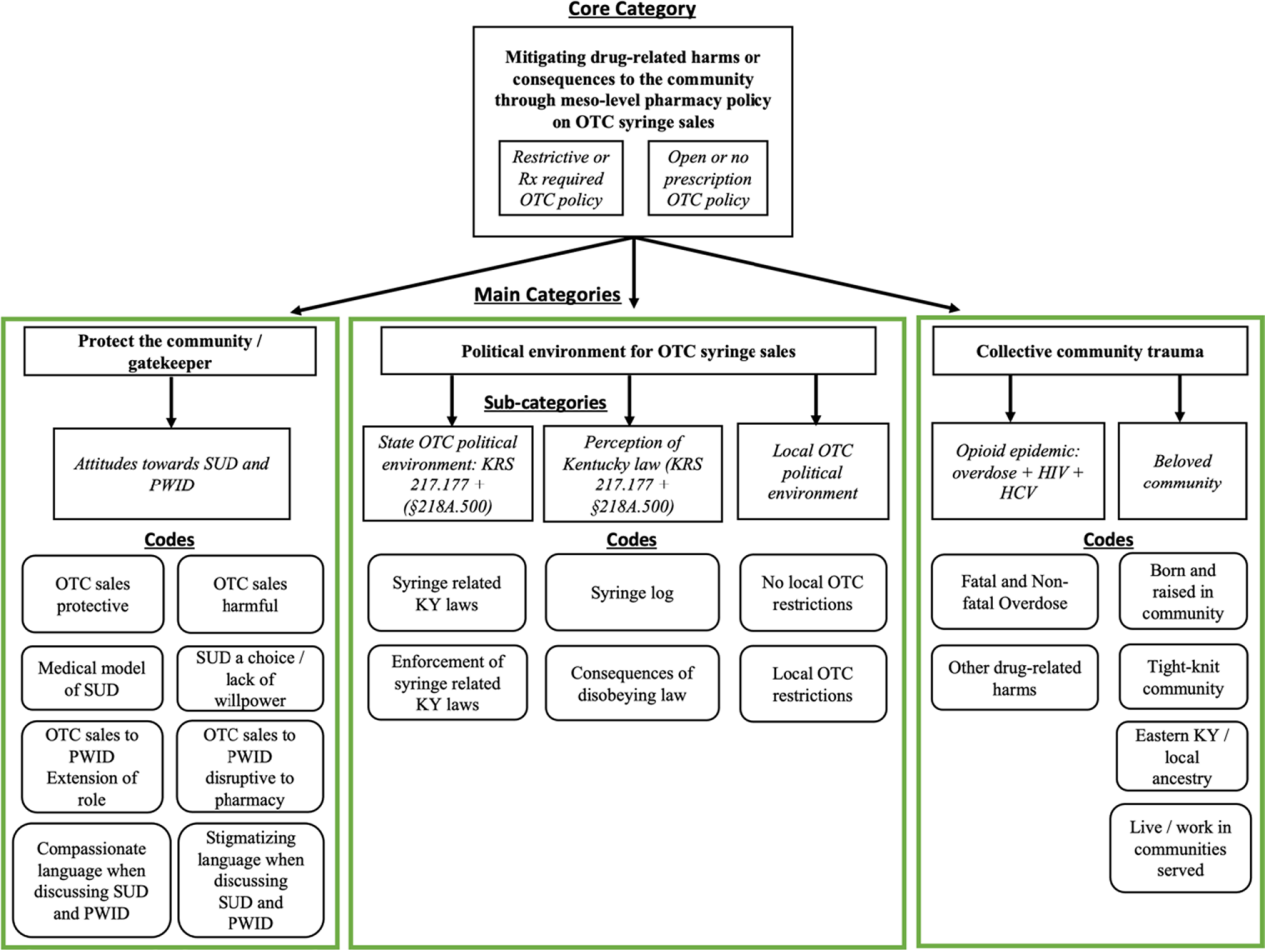
Visual overview of analytic categories, sub-categories and higher-order codes. The main categories and core category that arose from the grounded theory analysis are in bold text; *sub-categories* further describing and explaining the categories are in italic text; and finally, the higher-order codes, describing most salient concepts, are below the *sub-categories* in plain text

We followed traditional grounded theory steps. During open coding, two researchers, MF and HC, used line-by-line coding to develop the codebook. Coupled with sensitizing constructs, this method allowed themes or patterns in the data to arise organically rather than imposing a preconceived coding scheme. We used analytic memos and team discussions, to group codes into categories like “Protect the community,” and “Political environment for OTC syringe sales” and sub-categories like “Beloved community” that were internally coherent and mutually exclusive (Fig. 1). In the next stage, we used axial coding and the constant comparative method to explore relationships among categories and sub-categories. Finally, the selective coding stage identified the core category, “Mitigating drug-related harms or consequences to the community through meso-level pharmacy policy on OTC syringe sales,” and theoretical sampling was pursued to fully understand how this core category related to the others. Negative cases were sought to enhance validity. Transcripts were coded in NVivo 11. Disagreements were resolved through team discussions, until a consensus was reached on codes and categories.

One pharmacist operated two pharmacies in two different counties. OTC syringe sale policies differed across these two pharmacies, in part because county-level policies governing OTC syringe sales varied. We analyzed data on these two pharmacies separately.

### Ethics

Emory University's Institutional Review Board approved all data collection protocols for this study. Participants underwent a consent process prior to interviews.

## Results

Our analysis identified two groups of pharmacies, those that had restrictive OTC policies (“restrictive OTC pharmacies”) and those that did not (“open OTC pharmacies”) (Fig. 1). The analysis suggested that the following categories intersected within one another to influence whether pharmacists developed restrictive or open OTC syringe sale policies: “Protection of the community,” “Political environment for OTC syringe sales,” “Collective community trauma.” The core category that arose from our analysis was, “Mitigating drug-related harms or consequences to the community through meso-level pharmacy political on OTC syringe sales,” which intersected all other categories. Below we situate our categories from our grounded theory within the Risk Environment Framework, communicating our findings using REF domains.

### Overview and sample characteristics

Saturation was achieved with a sample of 14 pharmacists who operated 15 pharmacies, among which nine of twelve counties were represented. The sample was 2/3rds male, and all pharmacists identified as non-Hispanic White. The pharmacists in our sample represented 23% of all independent retail (i.e., non-hospital based) pharmacies located in the two health districts. Only one pharmacist worked in a retail pharmacy chain; other chain pharmacists either reported they had no time for an interview or were forbidden from participating.

### Pharmacy policy toward over-the-counter syringe sales

The analysis revealed two groups of pharmacies (Fig. 1): “restrictive OTC” pharmacies (*n* = 8) and “open OTC” pharmacies (*n* = 6). Pharmacies with restrictive policies required that patients provide proof of “legitimate medical need” before selling syringes OTC. Three of the eight restrictive pharmacies required that patients have a prescription for an injectable medicine and that they access that medication at the same pharmacy:

[I only allow people to purchase a syringe OTC] if they get insulin here. I don’t just sell needles over the counter.

Charlie*^1^

The other five “restrictive” pharmacies had policies that permitted some leniency if an unfamiliar patient could “reasonably convince” the pharmacist that they had a “legitimate medical need” for a syringe, which only included the injection of licit medications like insulin, B12, or testosterone:

…If you can tell me the type of insulin you take and [that] it’s something that you need to inject; yes, you can have some [OTC] syringes.

*Chris*

To assess “legitimacy,” restrictive pharmacies established protocols to vet unfamiliar patients. These protocols varied slightly but included one or more of the following: (1) asking the patient why they required syringes; (2) calling the patients’ home pharmacy to verify that they had a prescription for an injectable medication; and/or (3) asking the patient about their medication.

We have a process if need be. We occasionally [sell OTC syringes] to people if they are in need…We try to make sure that they have enough of a background info… usually at that point, if they can’t answer those questions pertaining to the medicine, then we know what’s going on.

*Alex*

In contrast, six pharmacies developed “open OTC sales” policies, in which they sold OTC syringes to almost any patient, as long as staff believed patients complied with Kentucky regulations,

As a general rule, [we’re] more than happy to sell syringes over the counter. You do legally have to provide a government-issued photo ID, and you do legally have to sign a syringe log that we keep behind the counter.

*Jordan*

In one case, an OTC pharmacist developed a policy that limited OTC syringes sales to patients who resided in the county where the pharmacy operated, as indicated on their ID,

...I do require that they live in the county. I don’t sell [OTC syringes] to people from out of town. They write their name, address, [and] show their ID. They put the reason for the purchase and how many they’re buying.

*Cameron*

**Fig. 2:**
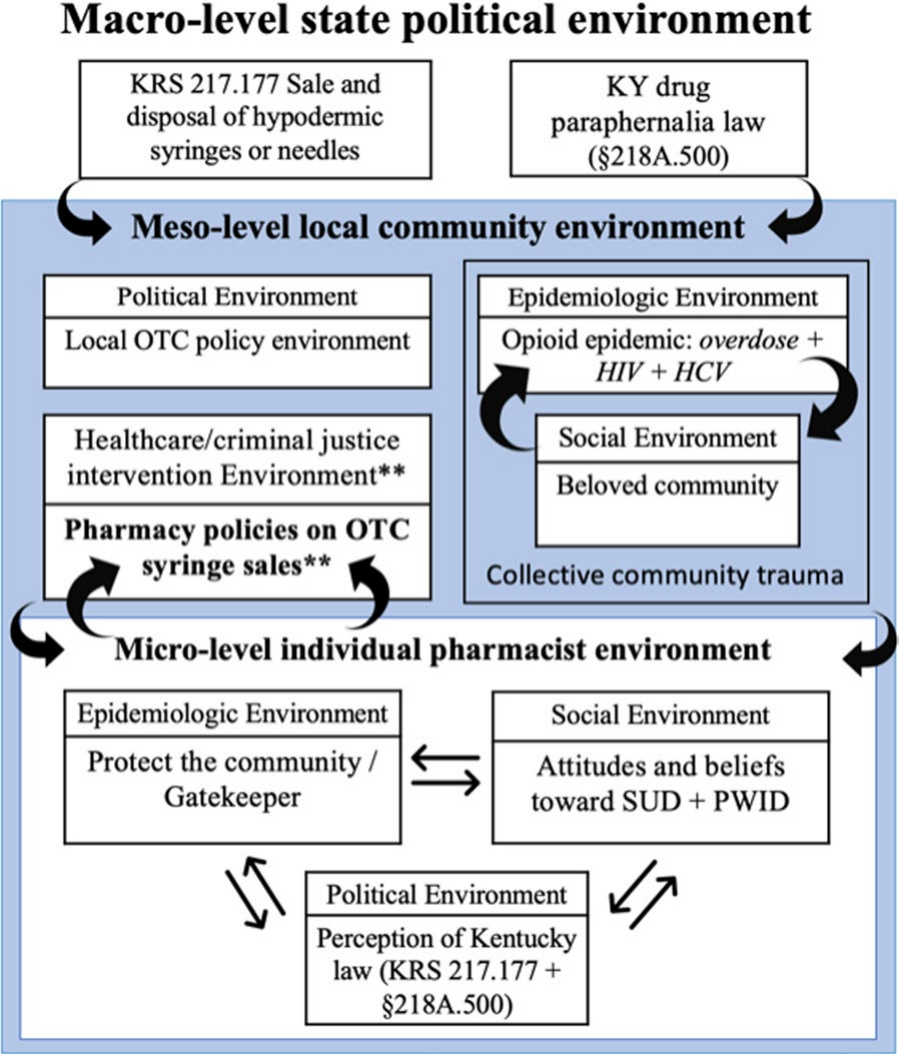
Visual overview of grounded theory integrated with risk environment framework (REF). We integrated our grounded theory with the Risk Environment Framework (REF); at the highest level, is the state-level political environment which influences and shapes the meso-level local community environment. Finally, the individual level pharmacist environment influences the meso-level health care/criminal justice intervention environment (outcome), while the other environments identified in the meso-level influence and shape the micro-level individual pharmacist environment

Our analysis indicates that these divergent OTC sales policies were formed through, and influenced by intersecting features of the macro-, meso-, and micro-level risk environments, summarized in Fig. 2, and explained in detail in the following sections.

### Impact of the opioid epidemic on the local community: collective community trauma

All pharmacies developed their OTC syringe sale policies to mitigate drug-related harms or consequences to their community, which was identified as the analysis’ core category, (Fig. 1). Close and long-standing social ties to their Eastern Kentucky communities were highly salient features of the meso-level social environment where pharmacists lived and practiced. These features and pharmacists’ connection to the area were captured by our sub-category, “beloved community” (Fig. 1). The majority of the pharmacists were born and raised in the area and reported familial roots dating back at least one generation,

[My family has been in this area] forever... My grandmother was from this area… So my whole family, generally, is just anchored to this area.

*Dorian*

Most pharmacists described the communities they served as “tight-knit,” “small town” places, where “everybody knows everybody” and where the people are, “good” and “trustworthy”:

I feel like it's a very tight-knit community…It's got a hometown feel…The people here of course are the greatest strength. People here are just different. They're just kinder and more willing to lend a helping hand…

*Carey*

Drug-related crises were features of the meso-level epidemiologic environment that scared and frustrated pharmacists by eroding their families and their broader communities. Though the interview guide did not query personal experiences with drugs, six pharmacists volunteered that they had friends and family members who struggled with substance use or died as a result of overdose,

…We do have a drug problem [that’s] increased over time…You don’t realize it until you try to step outside and look at just how bad it is…my children have lost friends to the drug problem. I’ve lost friends to the drug problem. I think it’s a deep problem.

*Morgan*

All pharmacists voiced multiple negative, far-reaching impacts of local drug-related epidemics, even if they did not discuss direct personal experiences.:

...[The opioid epidemic] affects everybody. People get so far gone, and…All they think about is how to get it. You can get robbed, your stuff stolen. It affects not just you, their family, their children. It affects everybody that they’re around and people that kind of aren’t even around them.

*Dylan*

Our analysis suggests that theses consequences interacted with pharmacists’ shared connection to the communities they served (beloved community), generating divergent OTC syringe sale policies, with divergences seemingly shaped by varied perceptions of the epidemiologic, social, and political environments (see Fig. 2). The following sections describe rationales for restrictive and open OTC syringe sale policies.

### Rationale for restrictive OTC syringe sale policies

Most pharmacists with restrictive policies wrestled with several barriers to OTC sales including (1) fear that OTC sales would harm their community or pharmacy; (2) stigma toward substance use disorder (SUD) and PWID, and (3) the need to strictly comply with their perception of Kentucky law. Though many ‘restrictive’ pharmacists wrestled with what the “right” decision was, they viewed themselves as gatekeepers and ultimately concluded that the potential harms to their community and pharmacy of selling OTC syringes outweighed the benefits of OTC sales.

Most pharmacists with “restrictive policies” were aware that sterile syringes reduced transmission of blood-borne infections, like HIV and Hepatitis C (HCV),

I think [sterile syringe access] would be an advantage for most patients because not sharing needles is going to decrease the spread of diseases like hepatitis.... [Hepatitis is preventable and Hepatitis infections are] very costly for the medical system...

*Chris*

However, they were concerned that selling syringes OTC would further harm their community and pharmacy by contributing to syringe littering in public spaces, “enabling” or “condoning” drug use, or disrupting their retail business and/or other customers,

The foot traffic [by PWID] is the worst thing about [selling syringes to PWID]... It’s just a group of people [you don’t want constantly on your premises]….we’ve got some of these elderly people that don’t need to be [exposed to PWID who act like heathens]…

*Jesse*

As illustrated by the previous quote, many of the concerns and rationale expressed by these pharmacists reflected fearful and stigmatizing views of SUD and PWID, a feature of the meso-level social environment. They often used stigmatizing language like “junkie,” “dirty” and “bad for business” to describe PWID. Some viewed PWID as “untrustworthy” and believed that they would discard their syringes inappropriately in public spaces, contributing to syringe littering, an intersecting feature of the epidemiologic and physical environment; or be disruptive to their other customers, an intersecting feature of the social and economic environment.

…[PWID] are going to use [the syringe] and then throw it down in the streets [or] in a park where the kids are going to go. Nobody wants that…You’re trusting a population who’s pretty much [not] trustworthy…I don’t know if [selling syringe to PWID] is good or bad for the community…only time will tell.

*Dorian*

The pharmacists’ rationale for their restrictive policies was further reinforced by an unclear state macro-level political environment that placed pharmacists in a difficult “grey area.” Kentucky law mandated that they had to maintain a log of all OTC sales that contained patient information including name, address and intended use for the purchased syringe. For restrictive pharmacists, sufficiently complying with the law meant ensuring that patients had “a legitimate medical use” for their syringes, which did not include injecting illicit substances like heroin,

Our logbook [requires] the patient say what they’re using [the syringe] for and it’s one of those things that I just can’t write something like “heroin” on the line, you know?

*Chris*

Some were concerned about the implications of frequent OTC sales:

I just don’t like someone’s name being on that syringe log a lot, especially when the Board of Pharmacy has to come in and look at it, like we’re haphazardly giving them out all the time. That’s really my barrier of what we have here.

*Jesse*

Pharmacists with restrictive OTC syringe sale policies assigned different weights to these various concerns, but ultimately viewed themselves as gatekeepers, protecting their community and pharmacy from escalating drug-related harms, seen as (1) syringe littering, (2) “enabling” or “condoning” drug use or (3) disrupting their retail business and/or other customers by attracting PWID to their pharmacies. Their rationale was further reinforced by their belief that restrictive policies were necessary to legally comply with Kentucky’s macro-level political environment (KRS 217.177 and KRS 218A.500).

### Rationale for open OTC syringe sale policies

Pharmacists with open OTC policies shared restrictive pharmacists’ concern for their community and pharmacy but had a more compassionate perspective; open OTC pharmacies believed that selling OTC syringes *protected* PWID and the broader community from harm. Their perspective was rooted in the belief that limiting syringes would not deter PWID from injecting drugs, and that access to sterile syringes decreased blood-borne infections:

[At first I restricted OTC syringe sales but] after a while, I became uncomfortable…[a PWID] is not going to not shoot up if they don’t have a clean syringe. They’re going to use a dirty syringe.

*Jordan*

They viewed selling syringes OTC to PWID as aligned with, or extending their health professional role, and desired to minimize harm to PWID:

I don't think you can change the world. But if you give [PWID] clean needles and things you can at least keep them healthy as you can keep them… and I think that fits in with trying to keep people healthy.

*Gerry*

Compared to pharmacists with restrictive policies, pharmacists with open OTC policies appeared to be less steeped in local stigma toward PWID, a feature of the meso-level local social environment. They expressed more patience and compassion for PWID and voiced a medicalized of view of SUD. Further, they did not express concern that PWID would disrupt their business or other customers.

Pharmacists with open OTC syringe sale policies believed they were protecting their community, including PWID, from escalating drug-related harms, like HIV and HCV. They did not express the same level of concern as restrictive pharmacists about syringe littering, “condoning” or “enabling” drug use or PWID disrupting their business and/or other customers. The state macro-level political environment was not mentioned as a barrier for pharmacists with open policies, and they did not mention attempting to “vet” patients seeking OTC syringes, beyond the requirements legally outlined in Kentucky law. This suggests OTC pharmacies believed (1) that sufficient compliance with state law only required asking for ID and completing the syringe log; and/or (2) they perceived the consequences for “selling paraphernalia” to be low or minimal, and they had a strong motivation to sell syringes OTC because it was “the right thing to do.”

### Negative case

We identified a negative case that did not align with our grounded theory: one pharmacist (Terry) reported that they could not sell syringes OTC because a local ordinance was in place that required all patients to have a prescription to purchase sterile syringes. This rule conflicted with their personal beliefs about OTC syringe sales: if it were not for the county law, they would sell syringes OTC:

“I would [sell syringes OTC] but [there’s a county law in place] that says patients have to have a prescription [for syringes]…”

*Terry*

## Discussion

Our qualitative analysis of pharmacists in two Eastern Kentucky health districts, an epicenter of the US opioid epidemic, found diverging OTC syringe sale policies, which we conceptualize as a feature of the meso-level health care/criminal justice intervention risk environment. Eight pharmacists had restrictive OTC syringe policies, requiring that patients provide proof of “legitimate medical need” to purchase an OTC syringe. Six pharmacists had open OTC syringe policies, which allowed most patients to purchase syringes OTC, provided the patient showed ID and supplied the information required to complete the syringe log.

Our study is the first qualitative study, to our knowledge, to examine pharmacist attitudes toward OTC syringe sales in a rural context in the USA. Our results echo and extend previous qualitative and quantitative work globally, in US cities and in the Appalachian region [19, 20, 23, 32, 36]. Similar to the other studies conducted in urban and rural areas globally, and to quantitative studies conducted in the US Appalachian South, we found that stigma toward SUD and PWID combined with restrictive laws governing drug paraphernalia and varied interpretations of these policies by pharmacists, to discourage pharmacy-based OTC syringe sales.

We build on previous studies such as Chiarello [9], Parry et al. [36] and Hagemeier et al. [20], by exploring the risk environment for OTC syringe sales in rural pharmacies, and by using qualitative data to enrich our understanding of the barriers and facilitators of OTC syringe sales in a rural context. We map our findings onto the REF (Fig. 2), finding that pharmacists experienced the opioid epidemic, a feature of the epidemiologic risk environment, as devastating their tightly knit, beloved communities, a feature of the meso-level social risk environment (Fig. 2). While pharmacists had a shared experience of community threat or trauma, divergent OTC syringe policies were rooted in varied perceptions of *which* threat they needed to protect their communities from. Pharmacists with restrictive OTC syringe policies were influenced by community stigma and believed that substance use and syringe littering were the primary threats to their community. To overcome these threats, they restricted PWID OTC syringe access. Conversely, pharmacists with open OTC sale policies perceived injection-related harms (e.g., HCV, HIV) as the primary threat and tried to counter it by getting sterile syringes into PWIDs’ hands. The political risk environment was also salient and further reinforced both groups’ policies and rationale; restrictive pharmacists were burdened by the syringe log, and intent on ensuring each patient had a “legitimate medical need,” aligned with KRS 217.177 and KRS 218A.500 (Fig. 2). Conversely, open OTC pharmacists did not perceive the pharmacy log as a barrier, suggesting that they believed that sufficient compliance with state law only required requesting ID and completing the syringe log, or that they perceived the benefits of doing the “right thing” outweighed the risk of legal consequences for “selling paraphernalia.”

Features of the risk environment identified in the current study that were similar to those found in urban areas included stigma toward PWID, a feature of the social environment, and macro- and meso-political related barriers, that caused hesitation among some pharmacies. However, at the time of our interviews, there were many features of the social, epidemiologic and the political risk environment that may be unique to these rural areas.

The social ties in rural areas, a salient feature of the social environment, have been found to be qualitatively different compared to metropolitan areas [15, 30, 46]. For example, the Pew Research Center found that 63% of rural residents surveyed reported living in their communities for eleven or more years, compared to 53% of suburban residents and 45% of urban residents [35]. One of our categories, the beloved community, highlights the involvement of the social risk environment and its centrality in co-producing the risk environment for syringe access. The pharmacists in our sample had lived in the area for decades; often their families lived in the area for generations. In these tight-knit communities, pharmacists frequently knew community members impacted by drug-related harms. Moreover, the severe and widespread nature of the opioid epidemic was unavoidable and was frequently described as “impacting everybody and everything.”

While differences in the strength, connectedness and structure of social ties in rural areas have been observed previously [15, 30, 46], we seek to center this feature of the social environment. As reflected in the language of the participants, the opioid epidemic is a shared and repeated collective trauma [3] that has devastated these already vulnerable communities [42]. We suggest that the differences between metropolitan and rural social environments impact the experience and consequences of collective trauma, creating the unique risk environment found in our study. Previous literature has noted how social ties in rural areas are stronger and more multiplex [1]; the smaller and more interconnected social networks of rural Appalachian residents compared to metropolitan residents, and generational family history in the area, mean that pharmacists intimately know community members suffering with OUD, dying from overdose and living with other drug-related consequences. We suggest that this social environment may be different from what is seen in cities, where social networks are larger, and individuals are more transient.

### Changes in the political environment and future implications

Effective July 1, 2021, the General Assembly of the Commonwealth of Kentucky enacted new statutory language officially relinquishing the syringe log requirement outlined in KRS 217.177 and adding language to KRS 218A.500 exempting pharmacies from paraphernalia restrictions. Additionally, the amendments to KRS 217.177 require pharmacies that sell syringes OTC to make available educational materials about safe syringe disposal, syringe exchange programs, substance use disorder treatment, as well as a verbal, physical or electronic offer for a naloxone prescription.

Previous studies suggest that evolving political environments may encourage OTC syringes sales among less enthusiastic pharmacists [9, 20]. By removing the log requirement for OTC syringe sales, more pharmacists may engage in OTC syringe sales. Our study suggests, however, that these macrolevel political changes may not be sufficient to transform pharmacists’ restrictive policies; while concerns about Kentucky law were salient for these pharmacists, they also viewed OTC sales as potentially harmful to the community and sought to gatekeep syringes from PWID. To shift these pharmacists’ OTC policies, additional anti-stigma education about the nature of SUD, injecting drug use, HIV, and HCV might be warranted. Additionally, testimonies from pharmacists engaged in OTC syringe sales might also shift attitudes and fears about the true impact of OTC sales on their pharmacy practice. Future studies should consider pre- and post-comparisons of pharmacy OTC sales, attitudes and practices among pharmacists in states where laws have changed.

### Limitations and strengths

We used Maxwell's framework to consider the study's validity [31]. Descriptive validity (i.e., the extent to which we captured what was said) was strengthened by using verbatim transcripts and comparing the transcripts to audio recordings. Interpretive validity (i.e., the extent to which the researcher captured participants’ meanings) was enhanced through extensive reflection, team discussions, and descriptive memoing. Theoretical validity was enhanced by a search for negative cases.

At the time of their interview, all but one pharmacist in our study worked at an independent pharmacy. The current study could not capture, and may not be generalizable to, policies and practices in a chain-based pharmacy setting. A previous study by Goodin et al. [19] in Kentucky found more chain pharmacies sold syringes without a prescription compared to independent pharmacies (71.5% vs. 51.9%, respectively). Their results suggested that barriers experienced and perceived by chain and independent pharmacies may be different and impact OTC syringe access [19].

## Conclusion

Expanding pharmacy-based access to OTC syringes is vital to curbing HIV and HCV epidemics in rural areas, in the USA and globally. While all pharmacists we interviewed developed their pharmacies’ OTC syringe policies to protect their beloved communities, more than half (*n* = 8) enacted policies that prohibited or greatly impeded OTC syringe sales. Our results suggest that the recent changes to Kentucky laws promoting OTC syringe sales may be insufficient to drastically shift restrictive policies, given that these organizational policies are also rooted in stigmatizing views of PWID and SUD. Legal changes should be accompanied by anti-stigma interventions designed to help currently restrictive pharmacists view HIV and HCV, rather than PWID themselves, as principal threats to their communities.

## Data Availability

Data sharing is not available for our qualitative dataset to protect participants privacy due to the sensitive nature of the data; there is risk that participants may be identified by their stories.

## Footnotes

1 *Pseudonyms used to protect participants confidentiality.
